# Efficacy of Myricetin Supplementation on Glucose and Lipid Metabolism: A Systematic Review and Meta-Analysis of In Vivo Mice Studies

**DOI:** 10.3390/nu16213730

**Published:** 2024-10-31

**Authors:** Mihai Babotă, Oleg Frumuzachi, Corneliu Tanase, Andrei Mocan

**Affiliations:** 1 Department of Pharmaceutical Botany, Faculty of Pharmacy, “George Emil Palade” University of Medicine, Pharmacy, Sciences and Technology of Târgu Mures, 540139 Târgu Mures, Romania; mihai.babota@umfst.ro (M.B.); corneliu.tanase@umfst.ro (C.T.); 2 Research Center of Medicinal and Aromatic Plants, “George Emil Palade” University of Medicine, Pharmacy, Sciences and Technology of Târgu Mures, 540139 Târgu Mures, Romania; 3 Department of Pharmaceutical Botany, “Iuliu Haţieganu” University of Medicine and Pharmacy, 400337 Cluj-Napoca, Romania; amocanm@gmail.com

**Keywords:** cardiometabolic health, 3,3′,4′,5,5′,7-hexahydroxyflavone, insulin resistance, dyslipidemia, antihyperglycemic, antihyperlipidemic, animal studies

## Abstract

Background/Objectives: Type 2 diabetes mellitus (T2DM) is a disorder characterized by insulin resistance, hyperglycemia, and dyslipidemia. Myricetin, a flavonoid found in various plants, has shown potential anti-diabetic effects in murine studies. This meta-analysis aimed to evaluate the impact of myricetin supplementation on glucose metabolism and lipid profiles in mouse models of metabolic diseases. Methods: A systematic review and meta-analysis were conducted in accordance with PRISMA guidelines (PROSPERO: CRD42024591569). Studies involving mice with metabolic disease models and exclusively using myricetin supplementation were checked across four databases (Embase, Scopus, PubMed, and WoS) until 23rd September 2024. The primary outcomes assessed were blood glucose (BG), insulin levels, triacylglycerol (TAG), total cholesterol (TC), HDL, and LDL. A random-effects model was applied to estimate standardized mean differences (SMD), and SYRCLE’s risk-of-bias tool for animal studies was used. Results: Twenty-one studies with 514 mice met the inclusion criteria. Myricetin supplementation significantly reduced BG (SMD = −1.45, CI: −1.91 to −0.99, *p* < 0.00001, *I*^2^ = 74%), insulin (SMD = −1.78, CI: −2.89 to −0.68, *p* = 0.002, *I*^2^ = 86%), TAG (SMD = −2.60, CI: −3.24 to −1.96, *p* < 0.00001, *I*^2^ = 81%), TC (SMD = −1.86, CI: −2.29 to −1.44, *p* < 0.00001, *I*^2^ = 62%), and LDL (SMD = −2.95, CI: −3.75 to −2.14, *p* < 0.00001, *I*^2^ = 74%). However, the effect on HDL was not statistically significant (SMD = 0.71, CI: −0.01 to 1.43, *p* = 0.05, *I*^2^ = 83%). Conclusions: Myricetin supplementation improved glucose metabolism and lipid profiles in mouse models, suggesting its potential as a therapeutic agent for managing T2DM. However, further research is needed to confirm these findings in human studies.

## 1. Introduction

Diabetes is a significant, long-term condition characterised by high blood glucose levels, linked to abnormal β-cell function affecting insulin activity [1]. The 2019 Global Burden of Diseases, Injuries, and Risk Factors Study (GBD) estimated that diabetes was the eighth leading cause of death and disability worldwide, affecting nearly 460 million individuals across all countries and age groups in 2019 [2]. Type 2 diabetes mellitus (T2DM) makes up around 90% of the estimated diabetes cases globally [1]. The risk factors for developing type 2 diabetes include high blood sugar levels, dyslipidaemia, insulin resistance, and excess body fat [3].

Polyphenols are a diverse group of compounds found abundantly in plants, where they serve as antioxidants and antimicrobial agents, protecting plants from UV light and pathogens. They can be divided into several classes, including flavonoids (such as quercetin, kaempferol, and myricetin), phenolic acids (like caffeic acid, ferulic acid, and gallic acid), stilbenes (including resveratrol, piceatannol, and pinosylvin), lignans (such as secoisolariciresinol, pinoresinol, and matairesinol), and tannins (like ellagitannin, procyanidin, and gallotannin) [4,5]. Consequently, flavonoids are categorized into six subclasses: flavanols or flavan-3-ols (including flavan-3-ol monomers, proanthocyanidins, and theaflavins), anthocyanidins, flavonols, flavanones, flavones, and isoflavones [6].

Numerous studies have demonstrated the health-promoting effects of these compounds when consumed, leading to their widespread use as functional dietary supplements and functional food components [7]. Strong evidence from epidemiological studies suggests that dietary polyphenols may help in managing and reducing the risk of developing T2DM [8]. Polyphenols from sources such as coffee, olive oil, and cocoa have been shown to exhibit anti-diabetic effects in T2DM patients by enhancing glucose metabolism, improving vascular function, and reducing insulin resistance and HbA1c levels [9,10,11,12].

**Figure 1:**
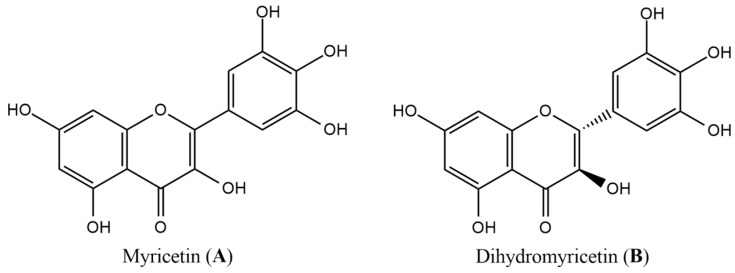
Chemical structures of myricetin (**A**) and dihydromyricetin (**B**).

Myricetin (Figure 1), a polyhydroxyflavonol, was first isolated from the bark of *Myrica rubra* (Lour.) S. et Zucc. This compound is abundant in various plant families, such as Myricaceae, Vitaceae, Leguminosae, Primulaceae, Rosaceae, Ericaceae, Fagaceae, and Compositae, and is commonly found in berries, fruits, vegetables, honey, red wine, tea, and other everyday foods [13]. Tea is the main source of myricetin in the human diet, accounting for 51.8% of intake, followed by wine (22.1%), coffee (8.9%), and vegetables (4.5%), with an estimated daily consumption of 2.3 mg [14].

Recent pharmacological research has demonstrated that myricetin exhibits several biological activities, including anti-inflammatory properties, anti-obesity effects, and cardiovascular protective benefits [15,16,17]. Epidemiological studies, including the EPIC-InterAct case-cohort study, found a strong association between myricetin intake and a reduced risk of T2DM, with myricetin showing the most pronounced inverse relationship among flavonols (such as isorhamnetin, kaempferol, and quercetin) [14]. A similar negative correlation between myricetin consumption and T2DM incidence was also observed in a Finnish study [18]. Although human intervention studies on myricetin are limited, the results have been promising. For example, supplementation with dihydromyricetin, a flavonoid structurally related to myricetin, at a dose of 600 mg/d in patients with non-alcoholic fatty liver disease (NAFLD) resulted in significant improvements in glucose and lipid metabolism, along with enhanced insulin sensitivity [19]. Likewise, another study involving supplementation with *Ampelopsis grossedentata* leaf extract, containing 970 mg of dihydromyricetin, showed improved glycaemic control in patients with T2DM [20]. The positive results seen in studies on (dyhydro)myricetin consumption and supplementation may be attributed to mechanisms such as the inhibition of carbohydrate digestion, the inhibition of glucose transport, and its action as a GLP-1 receptor agonist [21].

While many health benefits of myricetin are well-established, there is still a lack of human studies assessing the effects of pure myricetin supplementation. However, extensive research has been conducted on murine models, where myricetin supplementation has been found to stimulate lipogenesis in rat adipocytes and enhance insulin’s stimulatory effects. It also increased both enzymatic and non-enzymatic antioxidant defence systems in the liver and kidneys of a streptozotocin-cadmium-induced diabetic model, and reduced blood glucose levels by up to 50% within 2 days of treatment at a dosage of 3 mg/12 h [22].

Thus, the objective of this meta-analysis was to assess the impact of myricetin supplementation on health outcomes related to T2DM, including glucose metabolism (blood glucose (BG) and insulin levels) and lipid profile (triacylglycerol (TAG), total cholesterol (TC), high-density lipoprotein (HDL), and low-density lipoprotein (LDL) cholesterol) in mice (*Mus musculus domesticus* Schwarz and Schwarz) research models.

## 2. Materials and Methods

The meta-analysis was conducted following the Preferred Reporting Items for Systematic Reviews and Meta-Analyses (PRISMA-2020) guidelines to ensure transparency, accuracy, and thorough reporting. The study protocol was registered in the International Prospective Register of Systematic Reviews (PROSPERO) prior to beginning the review (CRD42024591569).

### 2.1. Eligibility and Exclusion Criteria

This meta-analysis focused on evaluating the effects of myricetin supplementation in in vivo experimental studies. The inclusion criteria were as follows: (i) only studies involving mice as the experimental subjects; (ii) mice had to be part of metabolic disease models, such as those induced for insulin resistance, hyperglycaemia, or hyperlipidaemia; (iii) studies were required to exclusively use myricetin supplementation as the intervention; (iv) studies must have reported fasting BG and insulin levels to assess glucose regulation and insulin sensitivity, and to measure TC, HDL- and LDL-cholesterol, and TAG levels to evaluate lipid profiles; (v) only controlled trials that included a control group (e.g., placebo or untreated) were considered; and (vi) the studies had to observe the short- or long-term (>5 d) effects of myricetin on glucose and lipid metabolism through longitudinal designs. The exclusion criteria were as follows: (i) studies involving species other than mice; (ii) studies that did not use metabolic disease models (e.g., insulin resistance, hyperglycaemia, and hyperlipidaemia) or used disease models unrelated to metabolism; (iii) studies that involved compounds other than or in addition to myricetin; (iv) studies that did not measure BG, insulin, TC, LDL-cholesterol, HDL-cholesterol, or TAG levels; (v) non-experimental studies, observational research, in vitro studies, human studies, and uncontrolled trials without a valid control group; and (vi) studies with intervention durations too short (minutes to hours) to observe meaningful effects or where the duration was not reported.

### 2.2. Search Strategy

Two researchers (M.B. and O.F.) independently conducted all phases of the search process. A thorough search was conducted across major databases, including Embase, Scopus, PubMed, and Web of Science, up to 23 September 2024, with no filters applied. The following search query was used: “(Myricetin AND (triglyceride OR triacylglycerol OR cholesterol OR low-density lipoprotein OR LDL OR high-density lipoprotein OR HDL OR glucose OR insulin) AND (in vivo OR mice)) NOT review”. Initially, the titles and abstracts of the retrieved articles were screened for eligibility. Full texts of relevant studies were then evaluated for inclusion, with any discrepancies between the researchers resolved through discussion and mutual agreement.

### 2.3. Data Extraction

Two researchers independently (M.B. and O.F.) performed the literature screening and data extraction, and then cross-verified the results. Any disagreements were resolved through discussion or by consulting a third researcher (A.M.). Using a standardized form, the extracted data included the following: (i) study characteristics (authors, year of publication, study design); (ii) details of the intervention (myricetin dosage, timing, route of administration); (iii) control conditions (type of control group, intervention details); (iv) methodological details (sample size, study duration, statistical methods used); and (v) outcome measures (BG, insulin, TAG, TC, HDL- and LDL-cholesterol).

### 2.4. Risk of Bias and Publication Bias Assessment

The SYRCLE’s risk-of-bias tool for animal studies was used to assess the risk of bias [23]. This tool includes 10 criteria associated with various types of bias, including selection bias, performance bias, detection bias, attrition bias, reporting bias, and other biases. The publication bias was assessed using the formal Egger’s test followed by trim-and-fill analysis.

### 2.5. Data Synthesis and Subgroup Analysis

Data analysis was performed using RevMan 5.4 software. Since data for all measured outcomes were continuous variables, the combined effects were reported as standardized mean differences (SMD) with 95% confidence intervals (CI), and a random-effects model was applied to the pooled data for the meta-analysis. Post-intervention mean values and standard deviations (SD) were extracted for both the intervention and control groups. When studies reported the standard error (SEM) as a measure of dispersion, the SD was calculated using the formula SD=SEM×√n, where “n” represents the number of subjects in each group. For studies presenting data in graphical format, numerical values were extracted using WebPlotDigitizer (https://automeris.io/, accessed on 27 September 2024). Heterogeneity was assessed using the *I*^2^ statistic, with heterogeneity considered present if the *I*^2^ value exceeded 50% or if there was inconsistency among the included studies. To explore potential sources of heterogeneity, a predefined subgroup analysis was conducted based on the intervention dose (<100 vs. 100–200 vs. >200 mg/kg) and duration (≤10 vs. >10 weeks (wk)). A sensitivity analysis was also performed to evaluate the impact of each study on the overall SMD.

## 3. Results

### 3.1. Search Results

**Figure 2:**
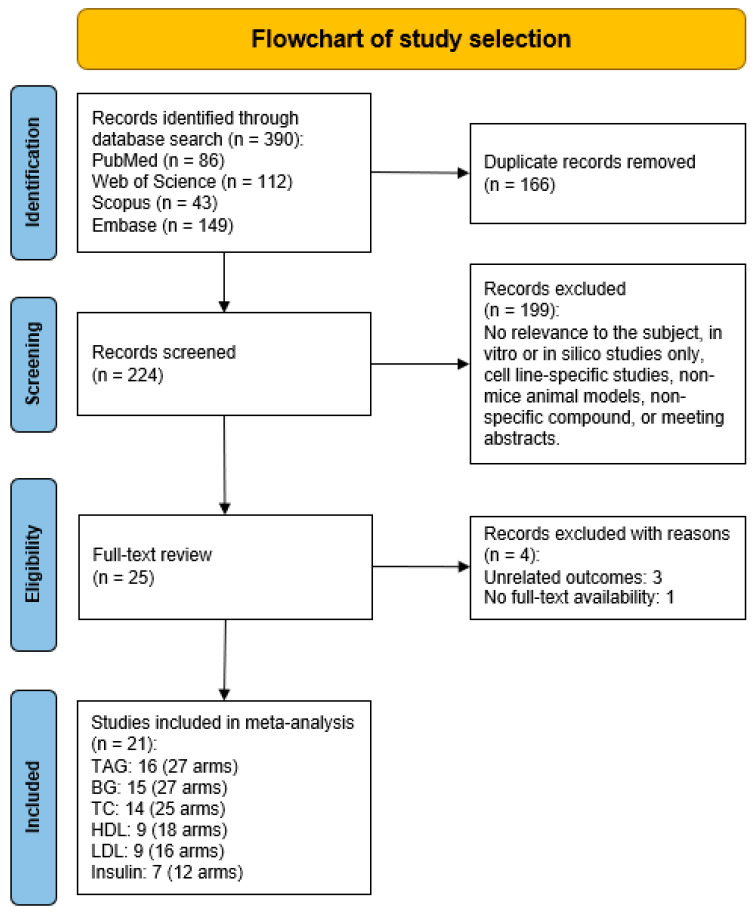
PRISMA flowchart illustrating study selection process for meta-analysis inclusion.

A total of 390 papers were initially identified through the database searches. After removing duplicates (n = 166), 224 papers remained for screening based on their titles and abstracts. Of these, 199 were excluded due to lack of relevance, in vitro or in silico studies, studies focusing on specific cell lines, use of non-mice animal models, non-specific compounds, or being meeting abstracts. This left 25 studies for further evaluation. An additional three records were excluded, with the reasons being unrelated outcomes (3) and lack of full-text availability (1). Ultimately, 21 studies were included in the meta-analysis: TAG (16 studies, 27 arms), BG (15 studies, 27 arms), TC (14 studies, 25 arms), HDL-cholesterol (9 studies, 18 arms), LDL-cholesterol (9 studies, 16 arms), and insulin (7 studies, 12 arms) (Figure 2).

### 3.2. Study Characteristics

The meta-analysis included a total of 514 mice, with 299 in the intervention groups and 215 in the control groups (Table 1). The interventions used varying doses of myricetin, ranging from a minimum of 5 mg/kg, as seen in Li et al. [23], to a maximum of 800 mg/kg, reported by Guo et al. [24]. The duration of interventions varied significantly from as short as one week (Li et al., 2024 [23]) to as long as 24 wk (Liao et al., 2017 [25]). A variety of animal strains were used across the studies, including C57BL/6J mice, db/db mice, *Ldlr*^−/−^ mice, Swiss mice, and Kunming mice. The experimental disease models included a broad range of conditions, such as diet-induced obesity and insulin resistance, NAFLD, leptin-deficient insulin resistance, and chronic vascular endothelial dysfunction. Many studies administered streptozotocin (STZ) to induce diabetic cardiomyopathy (DCM), diabetes, or diabetic nephropathy. Other models included doxorubicin (DOX)-induced cardiac injury, monosodium L-glutamate (MSG)-induced obesity, and choline-induced vascular dysfunction. The diets administered to these animals also differed, with some studies employing high-fat diets (HFD) or high-fat and high-sucrose (HFHS) diets, while others used standard mice chow or specialized diets such as the AIN-93G diet, Altromin HFD C1090, and the high-fat and high-cholesterol (HFHC) diet.

### 3.3. Risk-of-Bias Assessment

**Figure 3:**
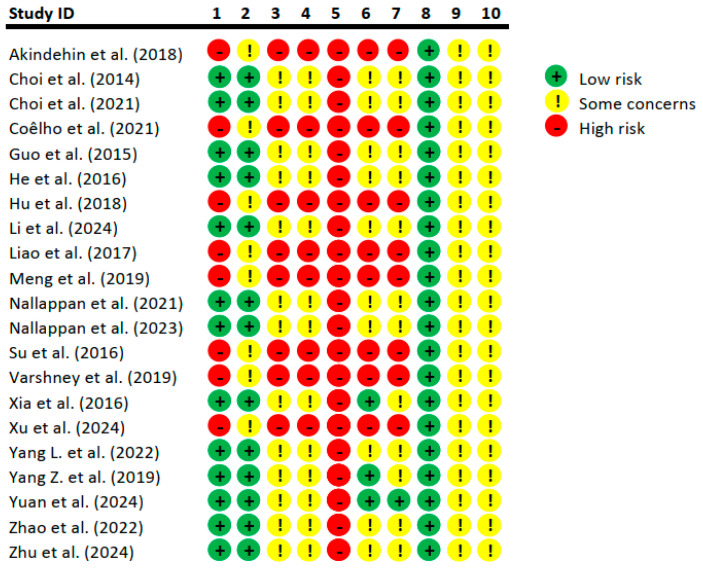
The risk of bias in the included studies evaluated using SYRCLE’s risk-of-bias tool for animal studies: sequence generation (1), baseline characteristics (2), allocation concealment (3), random housing (4), blinding of performance bias (5), blinding of detection bias (6), random outcome assessment (7), incomplete outcome data (8), selective outcome reporting (9), and other potential sources of bias (10), [16,24,25,26,27,28,29,30,31,32,33,34,35,36,37,38,39,40,41,42,43].

The risk-of-bias assessment for the included studies revealed several recurring concerns that potentially impacted the validity of the findings (Figure 3). Most of the studies exhibited a moderate-to-high risk of bias, largely due to incomplete reporting and deficiencies in key methodological aspects. One of the primary issues was the lack of clear information regarding the blinding of trial caregivers and researchers. In many cases, it was unclear whether caregivers and investigators were blinded to which intervention each animal received, increasing the potential for performance bias, where expectations or knowledge of treatment groups could influence the handling or assessment of the animals. Another significant issue was the incomplete description or inadequate implementation of randomization and allocation concealment processes. Randomization is crucial to ensure that animals are assigned to treatment and control groups in a way that minimizes selection bias. However, many studies did not provide sufficient details on how the randomization process was conducted, leaving room for potential bias in group assignment. Similarly, allocation concealment, which prevents researchers from knowing which group an animal will be assigned to before the intervention, was absent. This omission raises concerns about potential biases during the assignment phase of the experiment. Overall, the combination of unclear blinding practices and insufficient randomization and allocation concealment processes increased the risk of bias in many of the studies, potentially affecting the reliability and generalizability of their results.

The assessment of publication bias using Egger’s test and the trim-and-fill analysis raises important concerns regarding the validity of the meta-analysis results for most outcomes, particularly BG, TAG, TC, and LDL-cholesterol. The significant *p*-values in the asymmetry tests indicate that the observed effects may be influenced by the selective publication of positive results, meaning that the actual effects could be weaker than reported (Supplementary Materials, Table S1 and Figure S1).

### 3.4. Effects of Myricetin Supplementation on Glucose Metabolism

**Figure 4:**
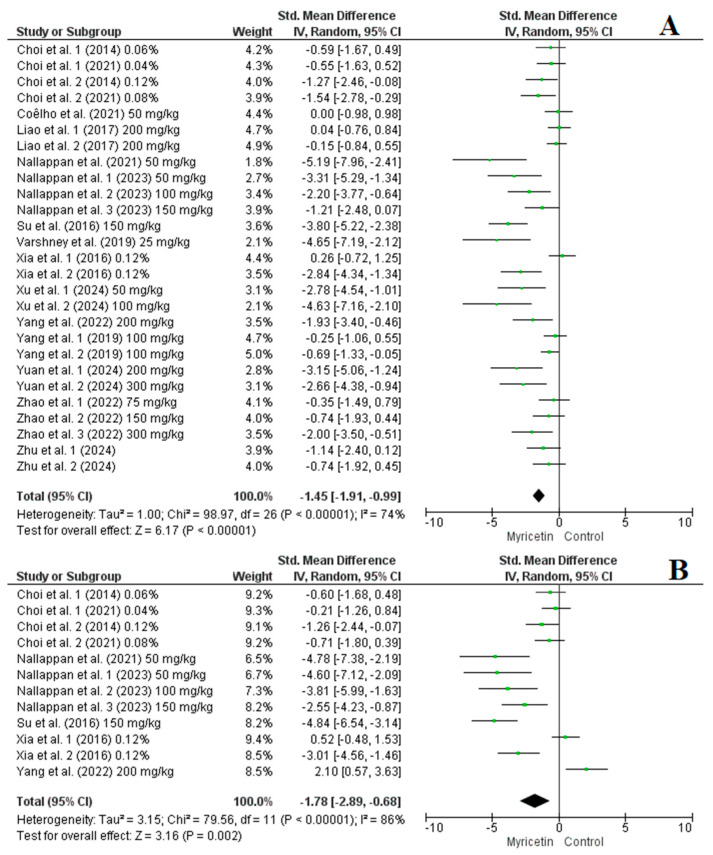
Forest plot representation of included studies evaluating the impact of myricetin supplementation on blood glucose (**A**) [25,27,28,29,32,33,34,35,36,37,38,39,40,41,42] and insulin levels (**B**) [27,28,32,33,34,36,38].

Among the studies included in the analysis, 15 studies with a total of 27 arms reported on the impact of myricetin supplementation on blood glucose levels, while 7 studies comprising 12 arms focused on insulin levels. The pooled results from the random-effects model indicated that myricetin supplementation led to a significant reduction in blood glucose (SMD = −1.45, CI: −1.91 to −0.99, *p* < 0.00001, *I*^2^ = 74%) (Figure 4A) and insulin levels (SMD = −1.78, CI: −2.89 to −0.68, *p* = 0.002, *I*^2^ = 86%) (Figure 4B) in the intervention groups. Subgroup analysis showed that myricetin supplementation positively affected blood glucose levels, regardless of dose (<100 mg/kg vs. 100–200 mg/kg vs. >200 mg/kg) or duration (≤10 wk vs. >10 wk), with no significant differences between subgroups (Supplementary Materials, Figure S2). However, for insulin levels interventions with doses of <100 mg/kg were more effective at reducing insulin levels compared to those with 100–200 mg/kg. Additionally, longer supplementation durations (>10 wk) were more effective at enhancing insulin sensitivity (Supplementary Materials, Figure S3). The sensitivity analysis did not significantly alter the results.

### 3.5. Effects of Myricetin Supplementation on Lipid Profile

**Figure 5:**
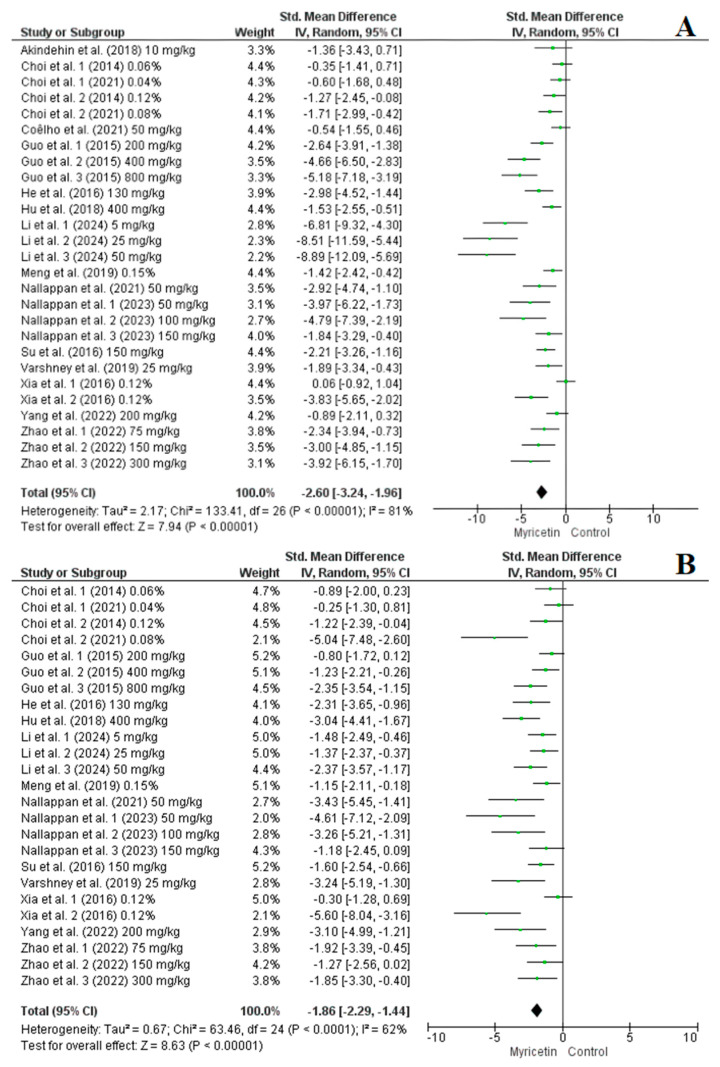
Forest plot representation of included studies evaluating the impact of myricetin supplementation on triacylglycerol (**A**) [16,23,24,26,27,28,29,30,31,32,33,34,35,36,38,41] and total cholesterol levels (**B**) [16,23,24,27,28,30,31,32,33,34,35,36,38,41].

**Figure 6:**
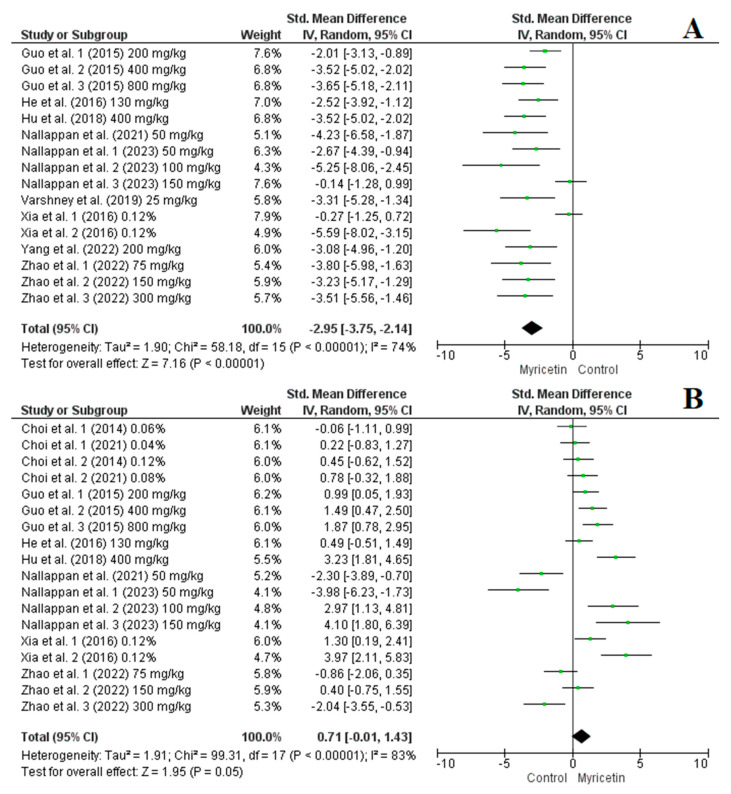
Forest plot representation of included studies evaluating the impact of myricetin supplementation on LDL-cholesterol (**A**) [16,24,30,32,33,35,36,38,41] and HDL-cholesterol levels (**B**) [16,24,27,28,30,32,33,36,41].

Among the studies analysed, 16 studies with 27 arms provided data on the effect of myricetin supplementation on TAG levels, 14 studies with 25 arms focused on TC, nine studies with 18 arms on HDL-cholesterol, and nine studies with 16 arms on LDL-cholesterol. The pooled results from the random-effects model showed that myricetin supplementation significantly reduced several lipid profile biomarkers: TAG (SMD = −2.60, CI: −3.24 to −1.96, *p* < 0.00001, I^2^ = 81%), TC (SMD = −1.86, CI: −2.29 to −1.44, *p* < 0.00001, I^2^ = 62%) (Figure 5, A and B, respectively) and LDL-cholesterol (SMD = −2.95, CI: −3.75 to −2.14, *p* < 0.00001, I^2^ = 74%) (Figure 6A). However, the effect on HDL-cholesterol was not statistically significant (SMD = 0.71, CI: −0.01 to 1.43, *p* = 0.05, I^2^ = 83%) (Figure 6B). Nevertheless, sensitivity analysis indicated that excluding the first arm of the Nallappan et al. (2023) study [33], which received 75 mg/kg of myricetin, led to a significant improvement in HDL-cholesterol levels (SMD = 0.90, CI: 0.23 to 1.58, *p* < 0.00001, I^2^ = 81%). The sensitivity analysis did not notably change the results for TAG, TC, or LDL-cholesterol levels.

Subgroup analysis showed that myricetin supplementation had a positive impact on TAG, TC, and LDL-cholesterol levels, regardless of dose (<100 mg/kg vs. 100–200 mg/kg vs. >200 mg/kg) or duration of intervention (≤10 wk vs. >10 wk), with no significant differences between subgroups (Supplementary Materials, Figures S4, S5, and S6, respectively). However, for HDL-cholesterol, the analysis revealed that only doses of 100–200 mg/kg significantly improved its levels (SMD = 1.45, CI: 0.38 to 2.52, *p* = 0.008, *I*^2^ = 70%), while doses above 200 mg/kg did not result in significant improvements (SMD = 1.17, CI: −0.69 to 3.02, *p* < 0.00001, *I*^2^ = 89%). Contrary to this, doses below 100 mg/kg significantly reduced HDL-cholesterol (SMD = −2.17, CI: −3.86 to −0.49, *p* = 0.01, *I*^2^ = 68%). Additionally, subgroups where myricetin was administered for longer than 10 wk showed significant improvements in HDL-cholesterol (SMD = 1.49, CI: 0.05 to 2.93, *p* = 0.04, *I*^2^ = 87%), compared to interventions lasting 10 wk or less (SMD = 0.21, CI: −0.54 to 0.95, *p* = 0.59, *I*^2^ = 77%) (Supplementary Materials, Figure S7).

## 4. Discussion

Dysregulation of glucose and lipid metabolism is a major contributor to the development of various chronic diseases, including T2DM, obesity, and cardiovascular disorders. A meta-analysis examining various polyphenol subclasses found that increased dietary consumption of flavanols, flavonols, flavan-3-ols, and isoflavones was significantly associated with a reduced risk of developing T2DM [8]. Another meta-analysis investigating the effects of flavonol supplementation on cardiovascular disorder biomarkers found significant improvements, including reductions in TC, LDL-cholesterol, TAG, BG, and blood pressure, along with an increase in HDL-cholesterol [43]. In the present meta-analysis, it was found that myricetin supplementation led to significant effects on both glucose metabolism and lipid profiles across various mice studies. In terms of glucose metabolism, myricetin supplementation led to a substantial reduction in BG and insulin levels. Subgroup analyses revealed that the positive effects on BG were consistent across all doses and durations of myricetin supplementation, without significant differences between subgroups. However, for insulin levels, lower doses (<100 mg/kg) were more effective compared to doses of 100–200 mg/kg, and longer supplementation durations (>10 wk) enhanced insulin sensitivity more than shorter supplementation durations (≤10 wk).

Regarding the lipid profile, the pooled results indicated that myricetin supplementation significantly reduced TAG, TC, and LDL-cholesterol levels (regardless of dose and duration), while the effect on HDL-cholesterol was not statistically significant. Subgroup analysis revealed that only doses of 100–200 mg/kg significantly improved HDL-cholesterol levels, while doses below 100 mg/kg significantly reduced HDL-cholesterol levels. Additionally, longer durations of myricetin supplementation (over 10 wk) showed significant improvements in HDL-cholesterol. Overall, myricetin supplementation appears to positively influence glucose metabolism and lipid profiles.

The findings presented align with existing literature, indicating that higher flavonol intake is associated with a lower risk of T2DM. For every 100 mg/day increase in flavonol consumption, the risk of T2DM drops by 3% [44]. Regarding specific flavonols, Tabrizi et al. demonstrated that quercetin supplementation significantly reduced TC and LDL-cholesterol in patients with metabolic syndrome but had no impact on TAG and HDL-cholesterol levels [45]. On the other hand, Guo et al. found that quercetin had no effect on plasma lipid levels in overweight and obese individuals, although it significantly reduced LDL-cholesterol at doses of ≥250 mg/day [46]. In animal studies, kaempferol (0.004% and 0.012% in drinking water, respectively) reduced fat weight, TC, and LDL-cholesterol while increasing HDL-cholesterol and improving glucose tolerance in mice. It also increased the expression of LDL receptor and apolipoprotein A1 genes, contributing to better lipid profiles. Also, kaempferol supplementation improved glucose tolerance which may be linked to lower serum resistin levels [47]. In HFD-fed rats, kaempferol (300 mg/kg) lowered body weight gain, visceral fat, plasma lipid levels, and hepatic TAG and cholesterol content. Additionally, it downregulated sterol regulatory element binding proteins (SREBPs), enhanced hepatic lipid metabolism, and increased acyl-CoA oxidase (ACO) and cytochrome P450 (CYP4A1) expression through peroxisome proliferator-activated receptor α (PPAR α) activation, suggesting kaempferol’s role in improving lipid metabolism and reducing fat accumulation [48].

Moreover, several model rat studies have investigated the impact of myricetin on blood glucose and lipid levels, indicating its potential benefits for metabolic health. For instance, Qian et al. found that myricetin supplementation (200 mg/kg body) in diabetic rats induced by a high-fat diet and STZ significantly reduced blood glucose and insulin levels while enhancing the expression of insulin receptor and glucose transporter 4 (GLUT4). The compound also protected pancreatic tissue, highlighting its positive effects on glucose metabolism and pancreatic health [49]. Similarly, Al-Abbasi and Kazmi demonstrated that co-treatment with myricetin and kaempferol (25 mg/kg) in STZ-induced diabetic rats improved glucose levels, lipid profiles, and inflammatory markers, showing a synergistic effect. This combination therapy reduced TAG, TC, and LDL-cholesterol while increasing HDL-cholesterol [50]. Kandasamy and Ashokkumar reported that myricetin (1.0 mg/kg) normalized glucose and insulin levels in STZ-cadmium (Cd)-induced diabetic nephrotoxic rats, improving carbohydrate metabolism and renal function markers. It enhanced insulin signalling through GLUT-2, GLUT-4, and protein kinase B (PKB), providing protection to liver, pancreas, and kidney tissues [51]. Liu et al. found that myricetin (3.0 mg/kg) improved insulin sensitivity and glucose utilization in fructose-fed rats by enhancing insulin receptor substrate-1 (IRS-1) and GLUT4 activity in muscle tissue, reversing insulin resistance and restoring normal insulin signalling [52]. Lalitha et al. showed that myricetin (20 mg/kg) reduced dipeptidyl peptidase-4 (DPP-4) activity, increasing glucagon-like peptide-1 (GLP-1) and insulin levels, and reducing NLRP3 inflammasome activation. Myricetin alone had a stronger effect on glucose control and antioxidant activity compared to when combined with horse gram (*Macrotyloma uniflorum* (Lam.) Verdc.) protein (100 mg/kg) [53]. Lastly, Chang et al. showed that myricetin supplementation (300 mg/kg) had anti-obesity and lipid-lowering effects by reducing body weight gain, visceral fat, and plasma lipid levels in HFD-fed rats, similar to fenofibrate (100 mg/kg). It decreased TAG and cholesterol content in the liver and lowered fat accumulation in liver cells and adipocytes. Mechanistically, myricetin upregulated the peroxisome proliferator-activated receptor α (PPARα), enhancing fatty acid oxidation, while downregulating SREBPs, which are involved in lipid synthesis. This dual action reduced body fat storage and promoted lipid breakdown, indicating myricetin’s potential in managing obesity and hyperlipidaemia [54].

Using in vitro model studies, Aminzadeh and Bashiri found that myricetin protected endothelial cells from high glucose-induced oxidative stress by enhancing antioxidant capacity and reducing markers of apoptosis, promoting cardiovascular health in diabetes [55]. Moreover, Karunakaran et al. confirmed that myricetin prevents pancreatic beta-cell dysfunction by inhibiting cyclin-dependent kinase 5 (CDK5) and oxidative stress, improving insulin secretion, beta-cell survival, and glucose-stimulated insulin release, which may protect against beta-cell failure in hyperglycaemic conditions [56,57]. Strobel et al. found that myricetin was able to modulate glucose metabolism through direct interactions with GLUT4, which is crucial for insulin-stimulated glucose uptake in adipocytes and muscles [58]. Finally, in a study investigating the effects of myricetin on atherosclerosis, it was found that myricetin significantly reduced macrophage accumulation in atherosclerotic lesions. Additionally, treatment with myricetin markedly decreased ox-LDL-induced cholesterol accumulation in macrophages, resulting in lower cellular cholesterol content and reduced lipid droplet formation. The reduction in cholesterol ester content further supports myricetin’s role in regulating lipid metabolism. Mechanistically, myricetin downregulated CD36 expression in macrophages, suggesting a pathway for its effects on lipid accumulation. Overall, these results highlight myricetin’s potential in modulating macrophage function and inflammation in atherosclerosis [31].

**Figure 7:**
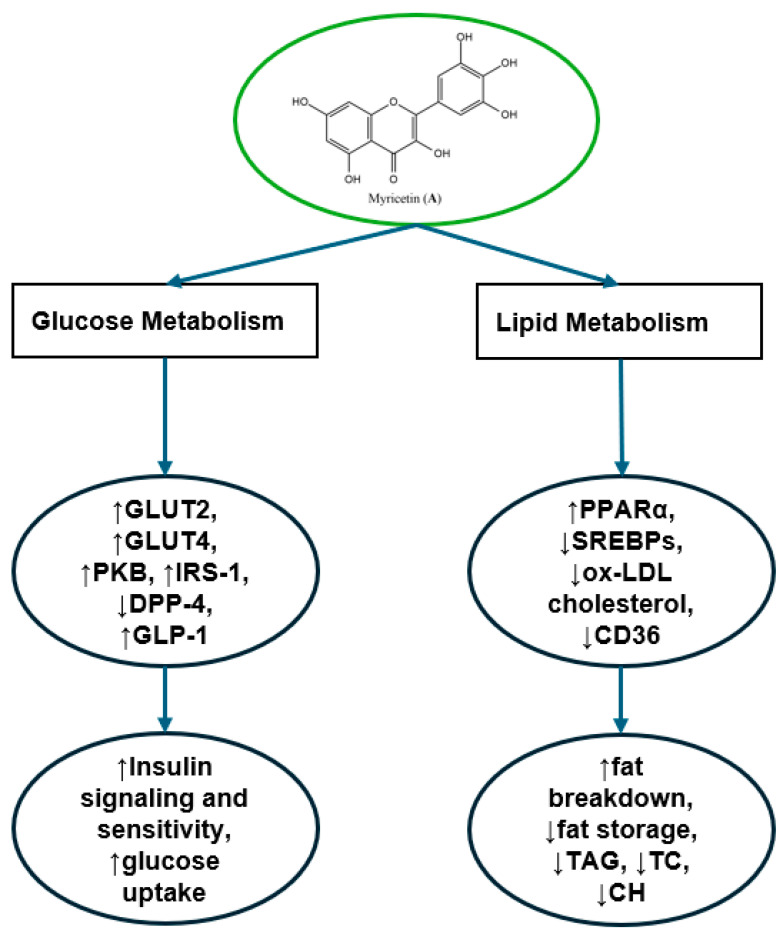
Molecular mechanisms of myricetin in regulating both glucose and lipid metabolism. On the glucose metabolism side, insulin sensitivity and glucose uptake are enhanced through the upregulation of key molecules such as GLUT2, GLUT4, PKB, and IRS-1, while DPP-4 is reduced and GLP-1 levels are increased. These actions lead to improved insulin signaling and glucose utilization. In lipid metabolism, PPARα is activated, SREBPs are suppressed, oxidized LDL (ox-LDL) cholesterol is reduced, and CD36 expression is downregulated, resulting in increased fat breakdown and reduced fat storage, triglycerides (TAG), total cholesterol (TC), and hepatic cholesterol (CH).

Taken together, these findings highlight the potential benefits of myricetin supplementation in addressing key T2DM risk factors, such as elevated blood sugar levels, insulin resistance, and dyslipidaemia (Figure 7). However, pharmacodynamic studies have identified several limitations. Myricetin’s oral bioavailability is low, around 9.62% and 9.74% at oral doses of 50 and 100 mg/kg, indicating poor absorption [59]. Additionally, its stability is affected by the gastrointestinal environment—it remains stable in simulated gastric fluids and low pH buffer solutions but undergoes pseudo-first-order degradation in simulated intestinal fluids and high pH buffer solutions [60]. Despite these challenges, the positive effects of orally administered myricetin have been consistently observed in cited animal studies. Moreover, two clinical trials with dihydromyricetin demonstrated improvements in glucose and lipid metabolism and enhanced insulin sensitivity [19,20], suggesting that myricetin could offer similar benefits in future human clinical trials.

## 5. Limitations

Despite the positive results reported by the present meta-analysis, several limitations should be acknowledged. The key one is that the analysis was based on post-intervention values, as the majority of the included studies did not report baseline data for the evaluated outcomes. This lack of baseline data limits a more precise understanding of the true effects of myricetin supplementation, as it prevents the assessment of changes from the baseline within the same experimental groups. Without this information, it is difficult to account for potential differences in baseline characteristics, which could influence the magnitude of the observed effects. Additionally, for studies that presented data in graphical format, numerical values were extracted using WebPlotDigitizer. While this tool is widely used for digitizing data from graphs, there is the potential for minor inaccuracies in the extraction process due to resolution and scaling issues, which could introduce a degree of variability in the reported effect sizes.

Also, the included studies in this meta-analysis exhibited high heterogeneity, as indicated by the *I*^2^ values for outcomes such as BG, insulin, TAG, and cholesterol levels. This variability is likely due to differences in study design, including intervention duration, animal models, and myricetin dosages. Although subgroup analyses were conducted, residual confounding factors may still affect the results. Moreover, there were few studies focusing on specific key outcomes like HDL-cholesterol and insulin levels, making the conclusions for these markers less robust. The significant heterogeneity in the HDL-cholesterol subgroup further emphasizes the need for additional research to confirm the reported findings. The risk-of-bias assessment uncovered several methodological concerns, with many studies lacking important details on blinding and randomization, which raises the potential for performance and selection bias. These weaknesses may undermine the reliability of the reported outcomes and potentially overstate the benefits of myricetin. Furthermore, publication bias was identified for multiple outcomes, particularly BG, insulin, TAG, TC, and LDL-cholesterol, suggesting that the meta-analysis could be influenced by selective reporting of positive results, leading to potentially inflated effect sizes.

Lastly, the results of this meta-analysis are derived solely from preclinical studies conducted on mice. While these findings provide valuable insights into the potential effects of myricetin on metabolic outcomes, caution is required when extrapolating these results to human populations. The only two human studies evaluating the impact of dihydromyricetin showed that it could improve glucose and lipid metabolism in subjects with metabolic diseases [19,20]. However, besides a narrow sample size (140 subjects in both studies) and the limited duration of these trials (1–3 months), the variability in dosing (600–970 mg/d) and population characteristics (T2DM and NAFLD subjects) limit the generalizability of these findings to a broader population. Additionally, it is important to highlight the differences between myricetin and dihydromyricetin, which may affect the interpretation of results. Although both compounds share similar structures, they may exhibit different pharmacological properties and metabolic effects. Nonetheless, these studies provide a useful starting point for further research, specifically in terms of dosing and expected outcomes. Therefore, further human clinical trials are necessary to confirm the efficacy and safety of myricetin supplementation in the context of metabolic diseases.

## 6. Conclusions

This meta-analysis provides evidence supporting the beneficial effects of myricetin supplementation on metabolic parameters, particularly in improving glucose regulation, insulin sensitivity, and lipid profiles in mice models of metabolic disease. Myricetin significantly reduced BG, insulin, TAG, TC, and LDL-cholesterol levels, with potential for a dose-dependent impact, especially for longer intervention durations. However, its effects on HDL-cholesterol remain inconclusive and require further investigation. Despite these findings, several limitations, including high heterogeneity, methodological weaknesses in the included studies, and potential publication bias, suggest that the overall effects may be overestimated. While the preclinical data are encouraging, translating these results to human populations demands cautious interpretation. Further well-designed clinical trials are necessary to validate the therapeutic potential of myricetin in managing metabolic diseases, such as T2DM. Nonetheless, this meta-analysis highlights myricetin’s potential as a valuable supplement for addressing key risk factors associated with cardiometabolic disorders.

## Figures and Tables

**Table 1:** Characteristics of included studies evaluating myricetin supplementation on outcomes related to T2DM, including glucose metabolism (blood glucose [BG] and insulin levels) and lipid profile (triacylglycerol [TAG], total cholesterol [TC], high-density lipoprotein [HDL], and low-density lipoprotein [LDL] cholesterol).

Study (Year), Country	Animals	Sex	Age (wk)	Experimental Model	Diet	Intervention		Duration (wk)	Sample Size			Measured Outcomes
						Myricetin Administration	Control		Myricetin	Control	Total	
Akindehin et al. (2018), Korea [26]	C57BL/6J mice	Male	6	Diet-induced obesity	HFD, D12492	10 mg/kg Intraperitoneally	Saline solution	2	3	3	6	TAG
Choi et al. (2014), Korea [27]	C57BL/6J mice	Male	5	Diet-induced obesity, insulin resistance	HFHS diet	0.06% (175 mg/d), 0.12% (325 mg/d) Dietary	–	12	7/7	7	21	BG, Ins, TAG, TC, HDL-C
Choi et al. (2021), Korea [28]	C57BL/6-Lep*^ob/ob^* mice	Male	4	Leptin-deficient, insulin resistance, NAFLD	AIN-93G diet	0.04%, 0.08% Dietary	–	10	7/7	7	21	BG, Ins, TAG, TC, HDL-C
Coêlho et al. (2021), Brazil [29]	Swiss mice	Male	3	MSG-induced obesity	Standard mice chow	50 mg/kg Orally	Distilled water	6	8	8	16	BG, TAG
Guo et al. (2015), China [24]	Kunming mice	Male	ND	Choline-induced chronic vascular endothelial dysfunction	Standard mice chow	200, 400, 800 mg/kg Orally	3% DCW, 0.5% CMC-Na	8	10/10/10	10	40	TAG, TC, LDL-C, HDL-C
He et al. (2016), China [30]	C57BL/6J mice	Male	3–4	Diet-infused hyperlipidemia	HFHC diet	130 mg/kg Orally	Distilled water	5	8	8	16	TAG, TC, LDL-C, HDL-C
Hu et al. (2018), China [16]	C57BLKS/J-Lepr*^db^*/Lepr*^db^* (db/db) mice	Male	4	Obesity, insulin resistance	Standard mice chow	400 mg/kg Orally	Distilled water	14	10	10	20	TAG, TC, LDL-C, HDL-C
Li et al. (2024), China [23]	C57BL/6J mice	Male	8	DOX-induced cardiac injury and dysfunction	Standard mice chow	5, 25, 50 mg/kd Orally	Saline solution	1	10/10/10	10	40	TAG, TC
Liao et al. (2017), China [25]	Mice	ND	ND	STZ-induced DCM	Standard mice chow	200 mg/kg Orally	Saline solution	24	12/16	12/16	56	BG
Meng et al. (2019), China [31]	*Ldlr*^−/−^ mice	ND	12	Dislipidemia and atherosclerosis	AsD diet	0.15% Dietary	–	8	10	10	20	TAG, TC
Nallappan et al. (2021), Malaysia [32]	C57BL/6J mice	Male	6	Diet-induced obesity, hyperglycemia, oxidative stress	Altromin HFD C1090	50 mg/kg Orally	Saline solution	4	6	6	12	BG, Ins, TAG, TC, LDL-C, HDL-C
Nallappan et al. (2023), Malaysia [33]	C57BL/6J mice	Male	6	Diet-induced obesity	Altromin HFD C1090	50, 100, 150 mg/kg Epigastrically	Saline solution	16	6/6/6	6	24	BG, Ins, TAG, TC, LDL-C, HDL-C
Su et al. (2016), China [34]	C57BL/6J mice	Male	4	Diet-induced obesity	HFD	150 mg/kg Orally	NS/Tween-80	10	12	12	24	BG, Ins, TAG, TC
Varshney et al. (2019), India [35]	C57BL/6J mice	Male	7	STZ-induced diabetes	HFD	25 mg/kg Intraperitoneally	10% ethanol: 40% PEG 400: 50% PBS	4	6	6	12	BG, TAG, TC, LDL-C
Xia et al. (2016), China [36]	C57BL/6J mice	Male	4	Diet-induced NAFLD	HFD	0.12% Dietary	–	12	8/8	8/8	32	BG, Ins, TAG, TC, LDL-C, HDL-C
Xu et al. (2024), China [37]	db/db mice	ND	6	Diabetic nephropathy	Standard mice chow	50, 100 mg/kg Intragastrically	Saline solution	12	6	6	12	BG
Yang L. et al. (2022), China [38]	C57BL/6J mice	Male	6–8	Diet-induced prediabetes	HFD, D12492	200 mg/kg Orally	Saline solution	8	6	6	12	BG, Ins, TAG, TC, LDL-C
Yang Z. et al. (2019), China [39]	C57BL/6J mice	ND	9–10	STZ-induced diabetes	Standard mice chow	100 mg/kg Orally	Distilled water	24	12/20	12/20	64	BG
Yuan et al. (2024), China [40]	C57BL/6J mice	Male	4	STZ-induced diabetic kidney disease	HFD	200, 300 mg/kg Intragastrically	Distilled water	11	6/6	6	18	BG
Zhao et al. (2022), China [41]	C57BL/6J mice	Male	3	STZ-induced diabetes	HFHS, D12492	75, 150, 300 mg/kg Orally	Distilled water	6	6/6/6	6	24	BG, TAG, TC, LDL-C, HDL-C
Zhu et al. (2024), China [42]	C57BL/6J mice	Male	4	STZ-induced DCM	HFD	ND Intragastrically	Vehicle	16	6/6	6/6	24	BG

Abbreviations: high-fat diet (HFD), high-fat and high-sucrose (HFHS) diet, non-alcoholic fatty liver disease (NAFLD), monosodium L-glutamate (MSG), dietary choline water (DCW), sodium carboxymethyl cellulose (CMC-Na), high-fat and high-cholesterol (HFHC) diet, doxorubicin (DOX), streptozotocin (STZ), diabetic cardiomyopathy (DCM).

## Data Availability

The original contributions presented in this study are included in the article/Supplementary Materials; further inquiries can be directed to the corresponding author.

## Supplementary Materials

The following supporting information can be downloaded at https://www.mdpi.com/article/10.3390/nu16213730/s1. Supplementary Table S1. The *p*-values for Egger’s test and meta trim-and-fill analysis for each outcome; Supplementary Figure S1. Funnel plots for selected outcomes: blood glucose (A), insulin (B), triacylglycerol (C), total cholesterol (D), HDL-cholesterol (E), and LDL-cholesterol (F); Supplementary Figure S2. Forest plot representation of included studies evaluating the impact of myricetin supplementation on blood glucose levels: subgroup analysis based on dose (A) and duration (B); Supplementary Figure S3. Forest plot representation of included studies evaluating the impact of myricetin supplementation on insulin levels: subgroup analysis based on dose (A) and duration (B); Supplementary Figure S4. Forest plot representation of included studies evaluating the impact of myricetin supplementation on triacylglycerol levels: subgroup analysis based on dose (A) and duration (B); Supplementary Figure S5. Forest plot representation of included studies evaluating the impact of myricetin supplementation on total cholesterol levels: subgroup analysis based on dose (A) and duration (B); Supplementary Figure S6. Forest plot representation of included studies evaluating the impact of myricetin supplementation on LDL-cholesterol levels: subgroup analysis based on dose (A) and duration (B); Supplementary Figure S7. Forest plot representation of included studies evaluating the impact of myricetin supplementation on HDL-cholesterol levels: subgroup analysis based on dose (A) and duration (B).
