# Supplementary material for: Efficacy of Myricetin Supplementation on Glucose and Lipid Metabolism: A Systematic Review and Meta-Analysis of In Vivo Mice Studies
Source: Nutrients. 2024 Oct 31;16(21):3730. doi: 10.3390/nu16213730 (PMC11547919; doi:10.3390/nu16213730)
Supplement: Supplementary file 1 [file nutrients-16-03730-s001.zip › nutrients-3276124-supplementary.pdf]

Supplementary Table S1. The p-values for Egger's test and meta trim and fill analysis for each outcome.

Outcomes	Test for Funnel Plot Asymmetry	Meta Trim and Fill Analysis
	p-value	
BG	< 0.0001	0.0134
Ins	0.0004	0.0518
TAG	< 0.0001	< 0.0001
TC	< 0.0001	< 0.0001
HDL	0.8477	0.1167
LDL	< 0.0001	< 0.0001

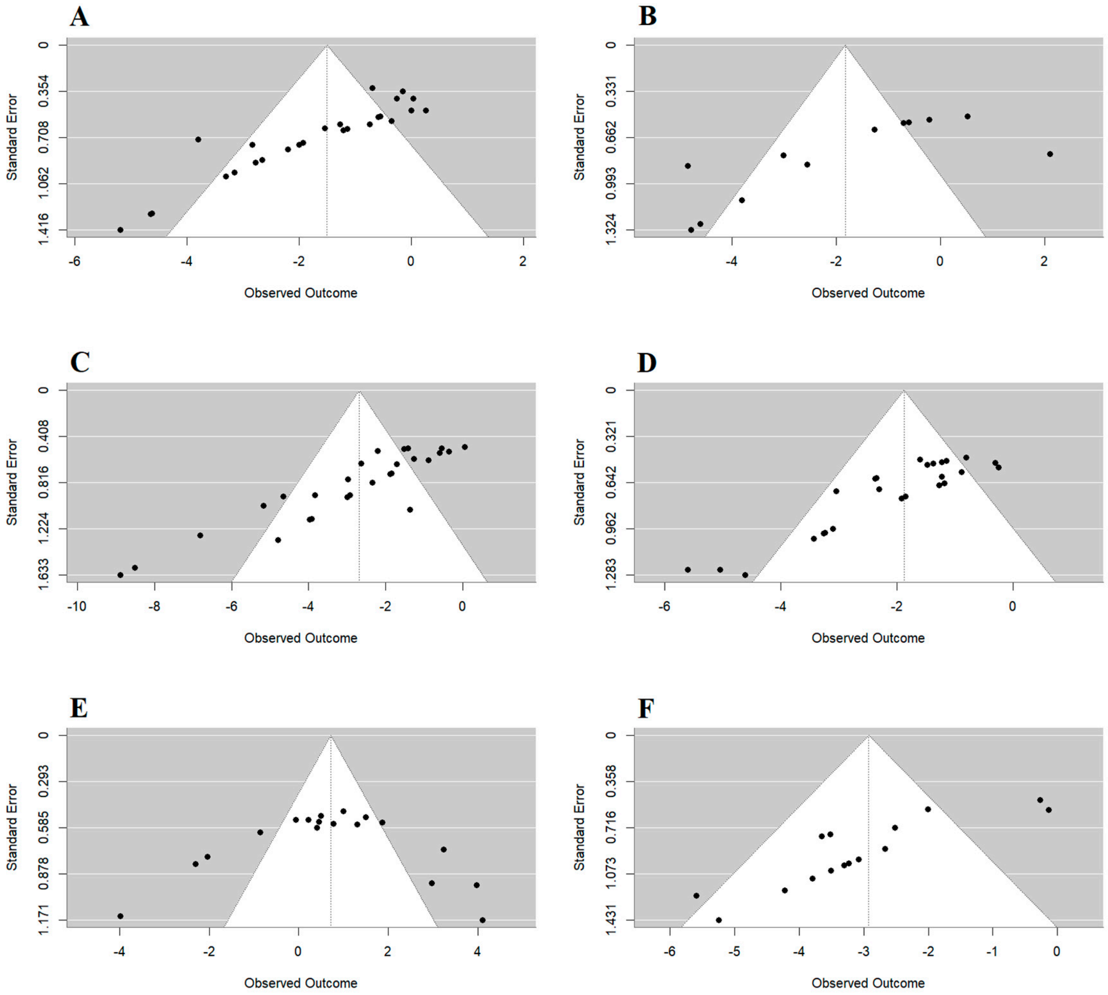

Supplementary Figure S1. Funnel plots for selected outcomes: blood glucose (**A**), insulin (**B**), triacylglycerol (**C**), total cholesterol (**D**), HDL-cholesterol (**E**), and LDL-cholesterol (**F**).

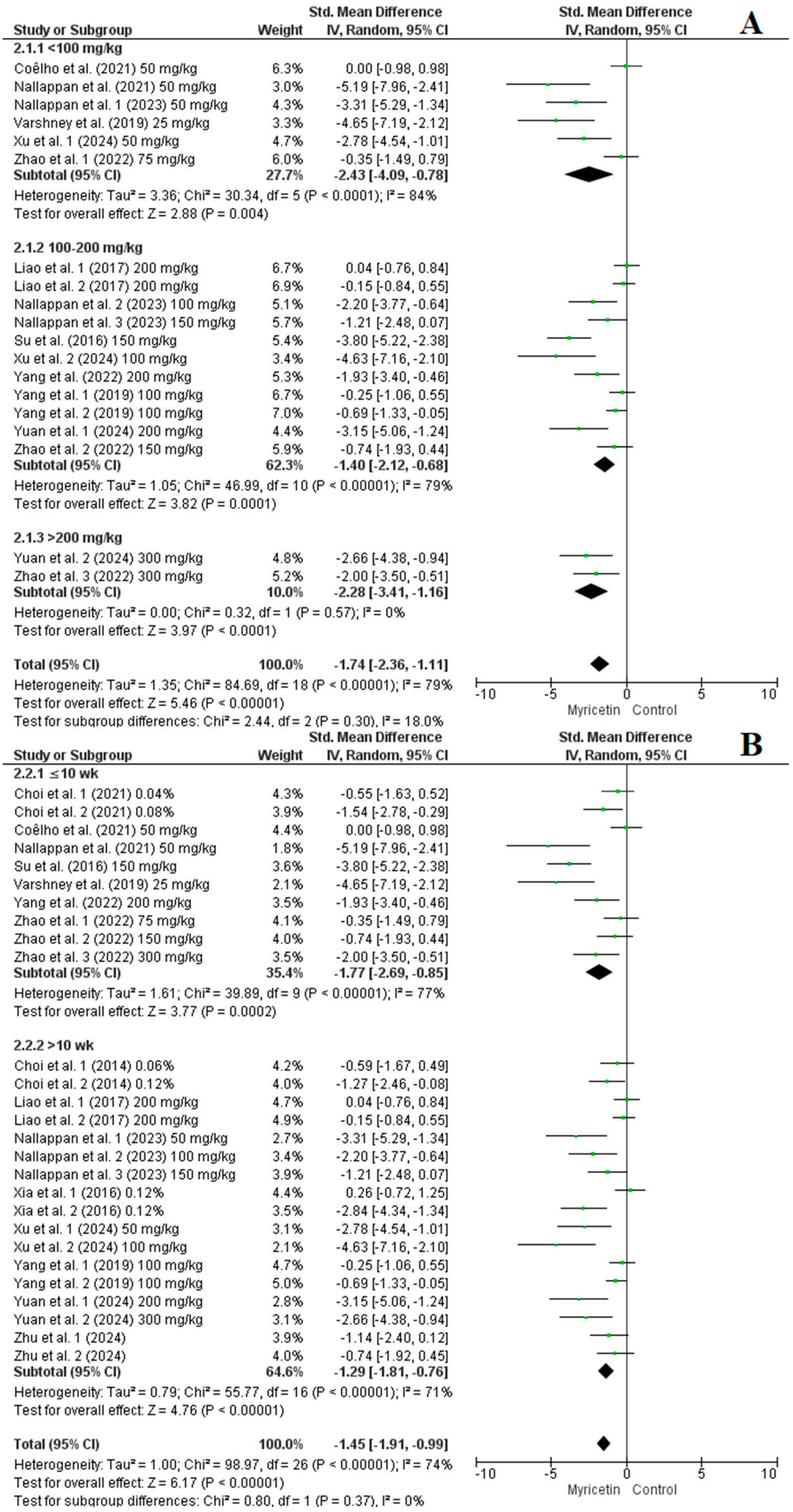

Supplementary Figure S2. Forest plot representation of included studies evaluating the impact of myricetin supplementation on blood glucose levels: subgroup analysis based on dose (**A**) and duration (**B**).

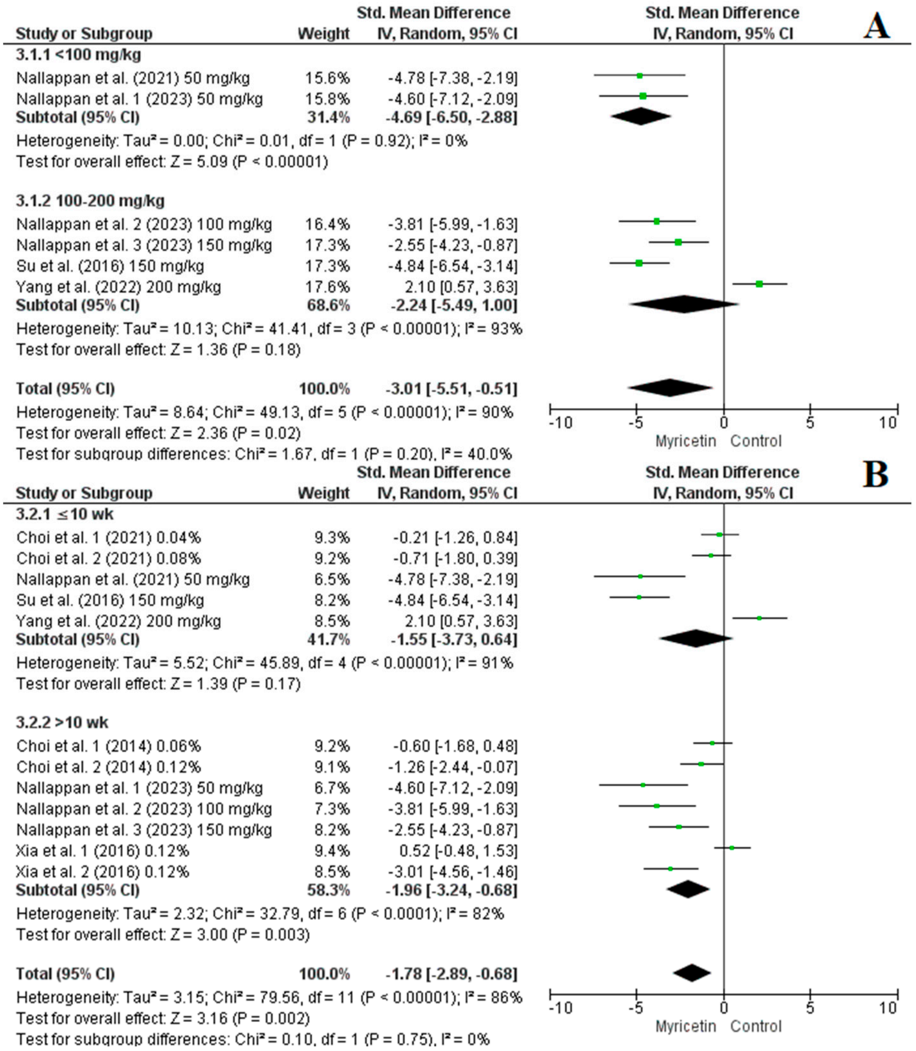

Supplementary Figure S3. Forest plot representation of included studies evaluating the impact of myricetin supplementation on insulin levels: subgroup analysis based on dose (**A**) and duration (**B**).

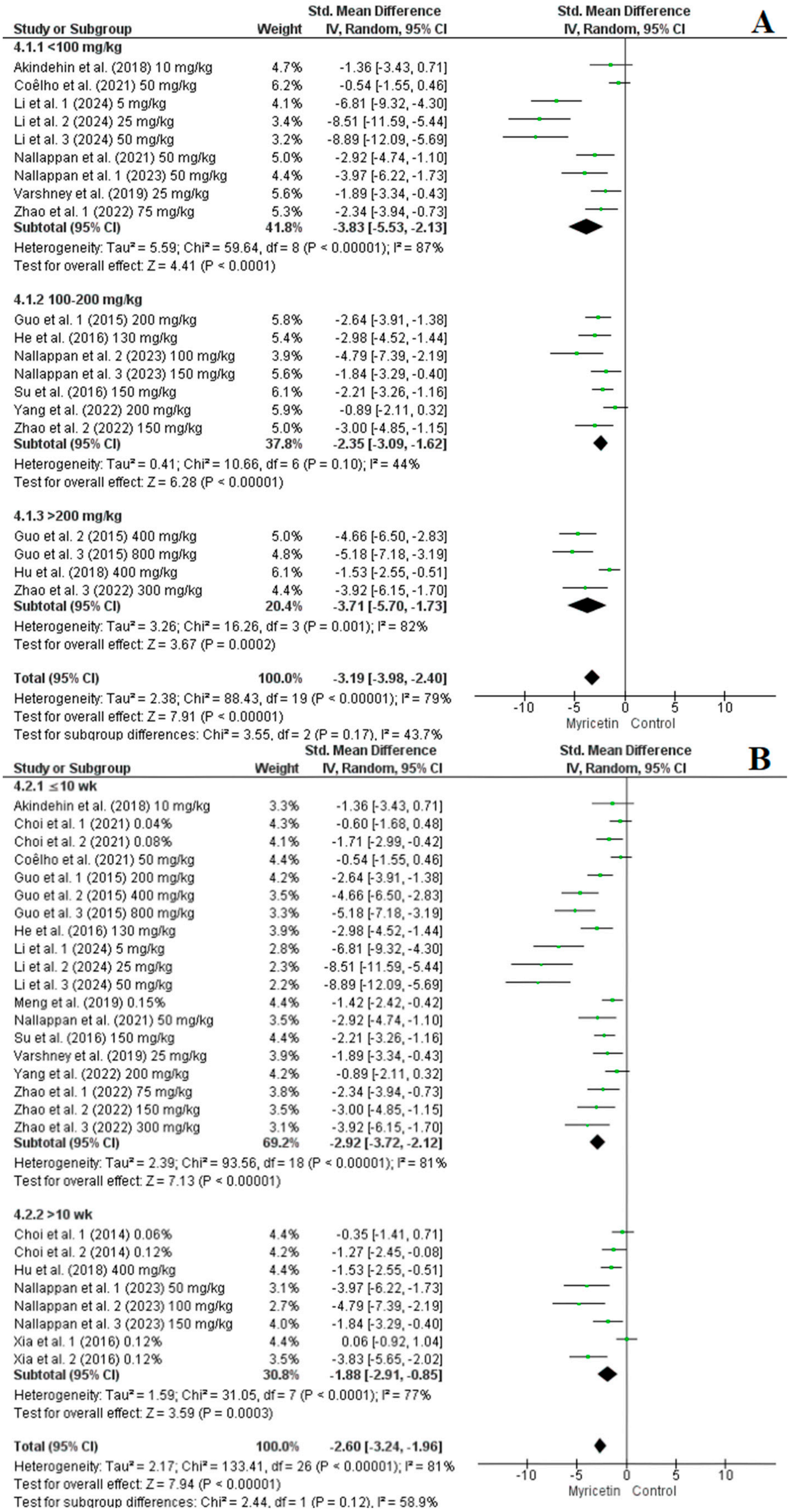

Supplementary Figure S4. Forest plot representation of included studies evaluating the impact of myricetin supplementation on triacylglycerol levels: subgroup analysis based on dose (**A**) and duration (**B**).

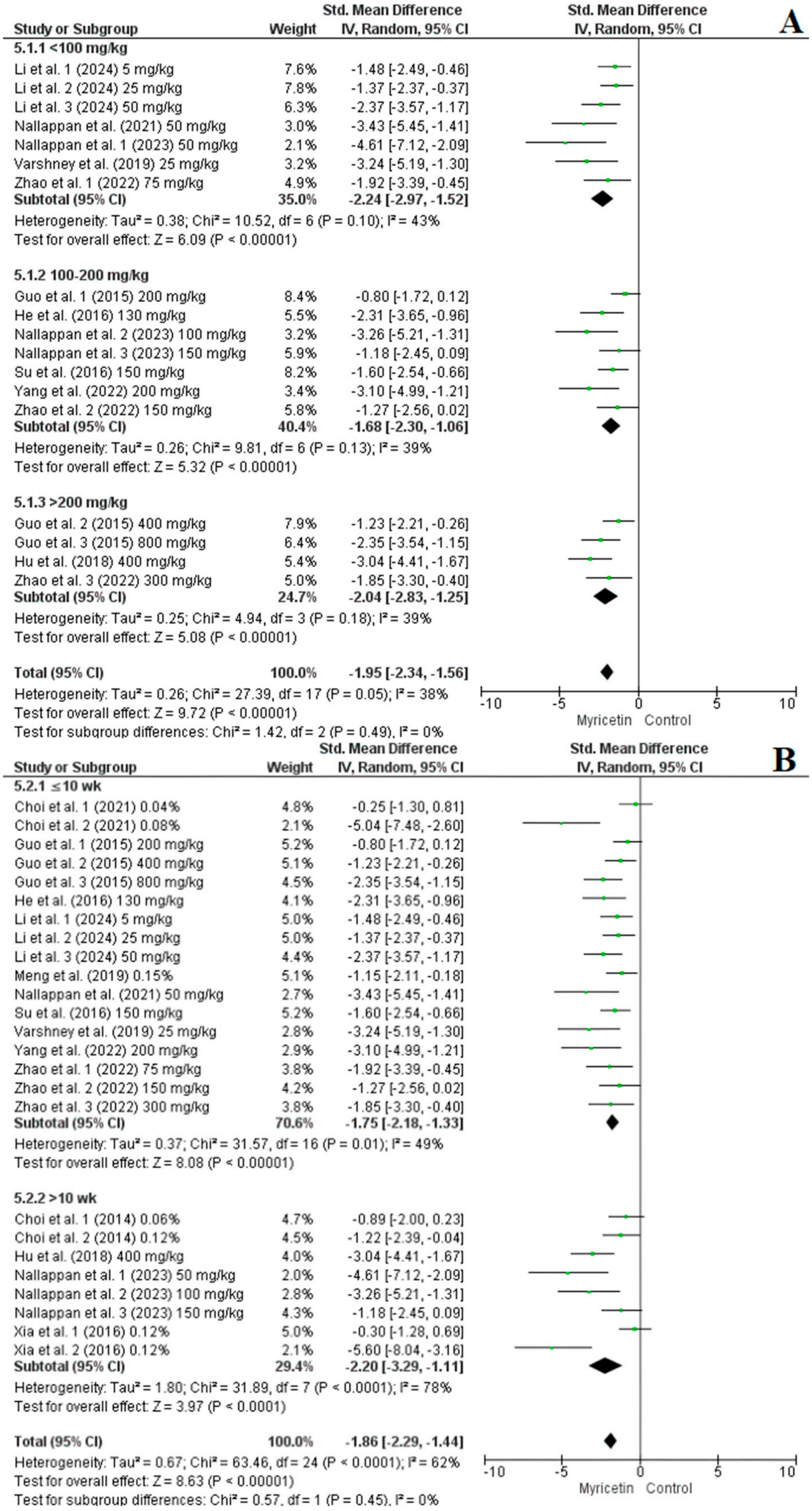

Supplementary Figure S5. Forest plot representation of included studies evaluating the impact of myricetin supplementation on total cholesterol levels: subgroup analysis based on dose (**A**) and duration (**B**).

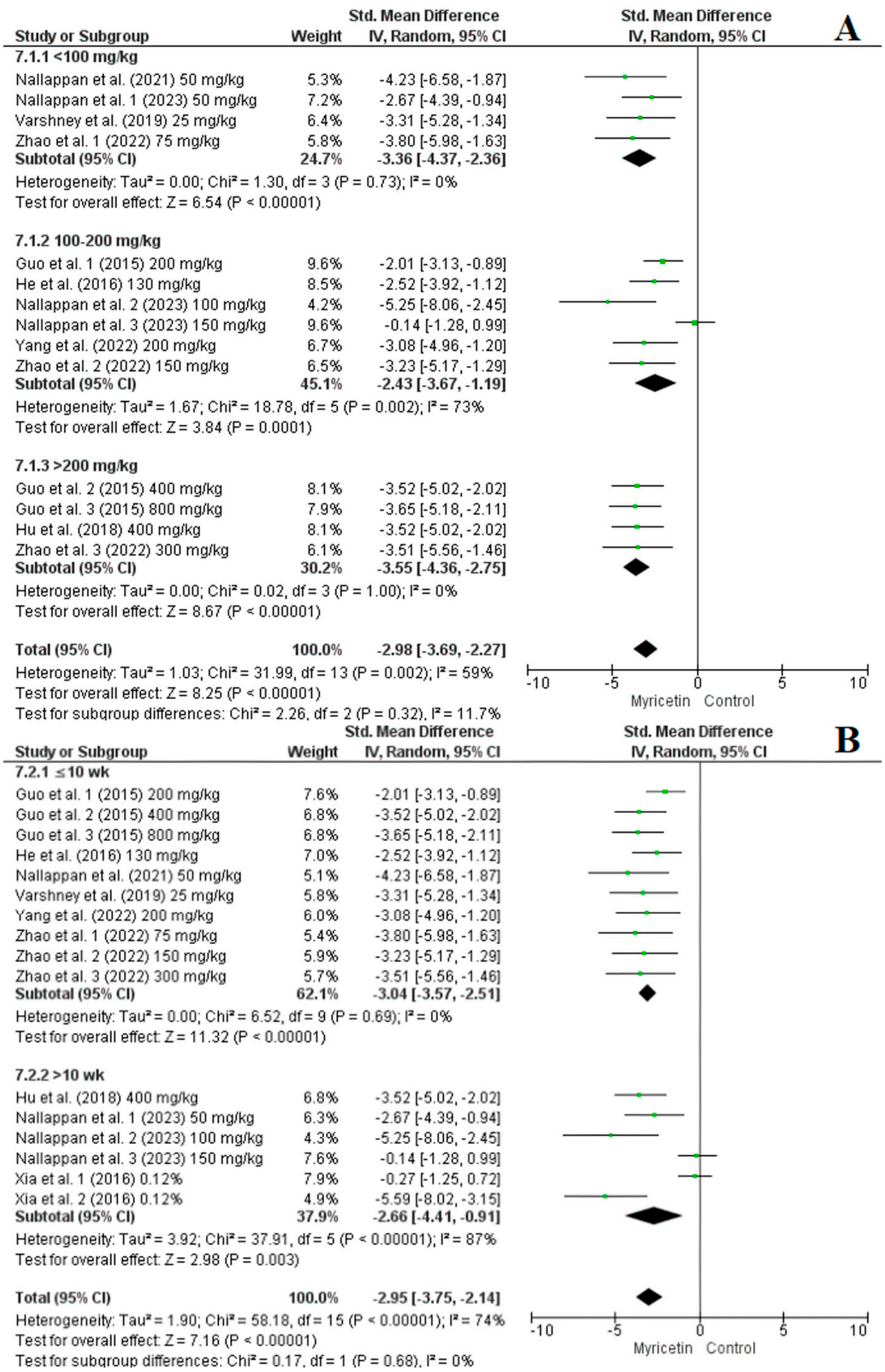

Supplementary Figure S6. Forest plot representation of included studies evaluating the impact of myricetin supplementation on LDL-cholesterol levels: subgroup analysis based on dose (**A**) and duration (**B**).

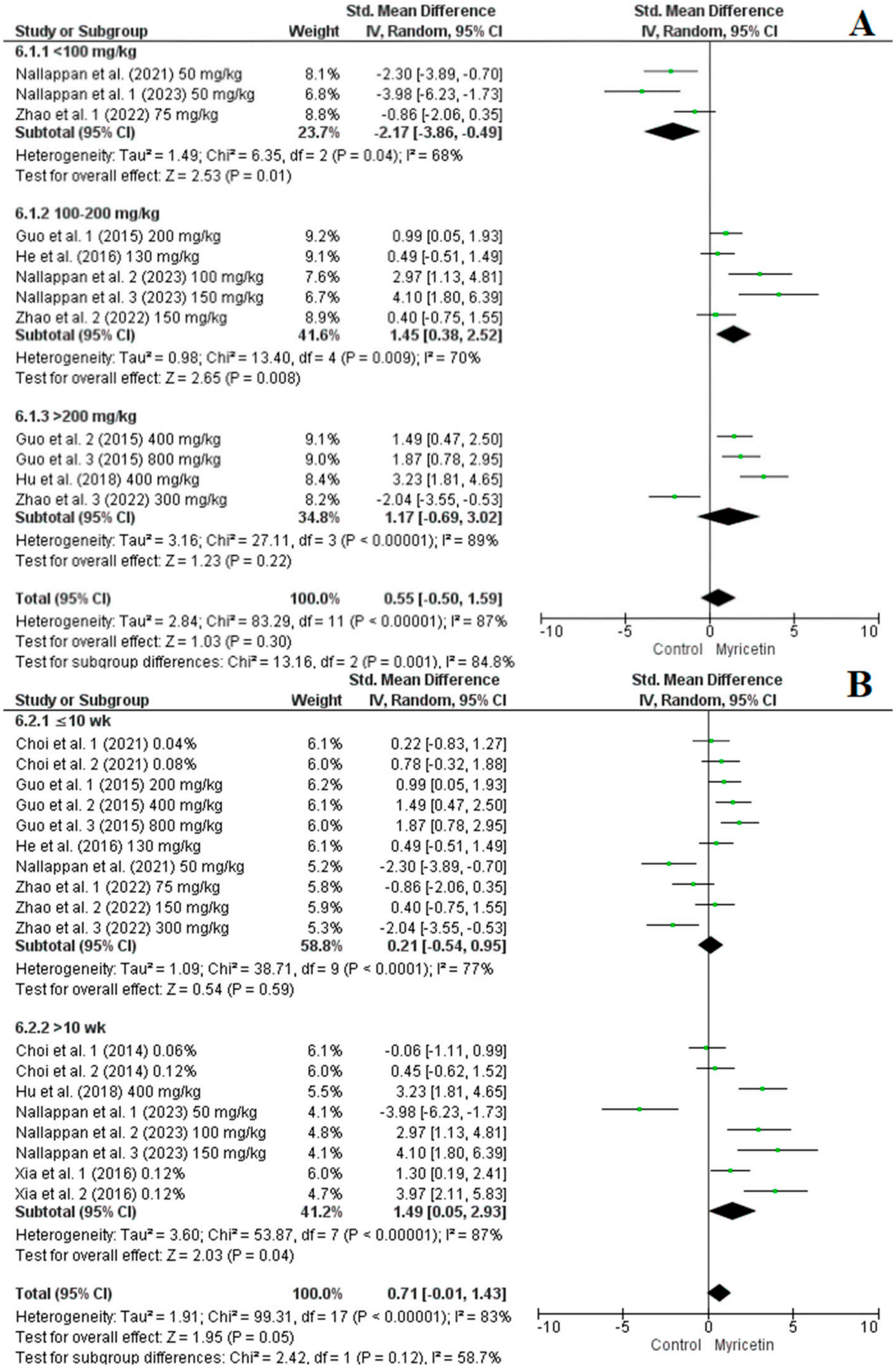

Supplementary Figure S7. Forest plot representation of included studies evaluating the impact of myricetin supplementation on HDL-cholesterol levels: subgroup analysis based on dose (**A**) and duration (**B**).
